# Organizational factors associated with readiness for change in residential aged care settings

**DOI:** 10.1186/s12913-018-2832-4

**Published:** 2018-02-01

**Authors:** Kathryn von Treuer, Gery Karantzas, Marita McCabe, David Mellor, Anastasia Konis, Tanya E. Davison, Daniel O’Connor

**Affiliations:** 1 Cairnmillar Institute, 391-393 Tooronga Road, Hawthorn, East Victoria 3123 Australia; 2 School of Medicine, Deakin University, Melbourne, Australia; 3 School of Psychology, Deakin University, Melbourne, Australia; 4 Institute for Health and Ageing, Australian Catholic University, Melbourne, Australia; 5 Department of Psychiatry, Monash University, Melbourne, Australia

**Keywords:** Organizational readiness for change, Organizational climate, Innovation, Transformational leadership, Aged care

## Abstract

**Background:**

Organizational change is inevitable in any workplace. Previous research has shown that leadership and a number of organizational climate and contextual variables can affect the adoption of change initiatives. The effect of these workplace variables is particularly important in stressful work sectors such as aged care where employees work with challenging older clients who frequently exhibit dementia and depression.

**Methods:**

This study sought to examine the effect of organizational climate and leadership variables on organizational readiness for change across 21 residential aged care facilities. Staff from each facility (*N* = 255) completed a self-report measure assessing organizational factors including organizational climate, leadership and readiness for change.

**Results:**

A hierarchical regression model revealed that the organizational climate variables of work pressure, innovation, and transformational leadership were predictive of employee perceptions of organizational readiness for change.

**Conclusion:**

These findings suggest that within aged care facilities an organization’s capacity to change their organizational climate and leadership practices may enhance an organization’s readiness for change.

**Electronic supplementary material:**

The online version of this article (10.1186/s12913-018-2832-4) contains supplementary material, which is available to authorized users.

## Background

The aged care sector is a highly dynamic and challenging workplace for both aged care managers and staff [1] who are required to provide high quality care to older adults with comorbid physical and complex mental health conditions [2, 3]. The sector is highly regulated and subject to policy changes with frequent revision of funding models [4]. These challenges are especially evident in residential aged care.

Within the last decade, aged care facilities across many countries have had to contend with the implementation of electronic medical record keeping, state and federal government changes to funding policies and care practices, and, more recently, a move to the person-centered care of residents [5–8]. The readiness of aged care staff to manage change is therefore likely to be an important factor affecting the operation of aged care facilities and the quality of care provided by staff to residents. Further, the capacity of workplaces and employees to change and introduce new programs, practices, or policies is central to continued organizational improvement [9]. However, little research has been conducted on the readiness of organizations for change within the context of aged care [10]. The factors that contribute to organizational readiness may well be very context specific.

Organizational readiness for change is a relatively new concept [11–13] that refers to the extent that employees will start or continue to engage in behaviors (such as support and participation) associated with change [14]. Holt, Armenakis, Feild and Harris [15] conceptualized organizational readiness for change as comprising four dimensions of readiness: appropriateness (i.e., employees perceive that the change is appropriate to the organization); managerial support (i.e., employees perceive that managers are supportive of the change); self-efficacy (i.e., employees perceive that they possess the skills and competencies to successfully cope with change); and personal valence (i.e., employees believe the change will be personally beneficial).

Despite the importance of organizational readiness for change within the aged care sector, only a few studies have sought to identify the contextual factors that may affect the success of a change initiative in this setting [10]. A greater understanding of these factors and their role in employee’s readiness to implement change initiatives should reveal how prepared aged care facilities are to undertake change, and how change might best be accomplished.

Research outside the aged care context has identified a range of organizational climate variables as important predictors of organizational readiness for change [16, 17]. Organizational climate is defined by the individual perceptions of their work environment and has been operationalized through measuring various factors, or subscales of the work environment [18]. There are many reported organizational climate variables which are typically determined through measuring levels of factors such as workplace autonomy, cohesion, trust, pressure, support, recognition, fairness, and innovation [18].

Specifically, organizational climate factors such as role clarity and innovation [19], commitment and trust have been found to be important antecedents to organizational readiness for change. McKay, Kuntz, and Naswell [20] found that organizational commitment was directly related to three of the four components of readiness for change; that is, perceptions of change as appropriate to the organization, change self-efficacy, and positive personal valence.

One of the few studies in a healthcare setting surveyed direct care workers (i.e., residential and child care workers) and clinicians (i.e., social workers, psychologists and guidance counselors) to determine how their agency’s organizational climate and job satisfaction influenced employee perceptions of agency readiness for change [19]. A number of organizational climate variables were measured. Structural equation modeling showed that role ambiguity, supervisor goal emphasis, organizational innovation, satisfaction with communication, and the number of years in their positions predicted workers’ perceptions of organizational readiness for change.

Leadership has also been found to play a central role in change readiness [20, 21] and it has been suggested that different styles of leadership are needed to affect the feelings (affects) and thinking (beliefs) components of change [22, 23]. More recent definitions of leadership highlight the importance of the relationship and influence between the leader and the followers and is reflected in inclusive definitions of leadership such as “…the reciprocal process of mobilizing persons … in order to realize goals …” [24]. Leadership is often conceptualized as two different styles of leadership, being transformational and/or transactional. By definition, transformational leaders are focused on change [25]. Transformational leadership motivates followers to identify with a leader’s vision and sacrifice their interests for that of the group or organization [26, 27]. Further, transformational leaders have been shown to generate awareness and acceptance among followers in relation to organizational missions and purposes resulting in a wide variety of positive outcomes [26, 27]. Thus, a transformational or adaptive style of leadership should increase employees’ feelings and beliefs in relation to the change and change process.

A leadership style that is transactional or exchange-oriented could also motivate employees’ readiness for change [28]. A transactional leadership style entails the explicit setting of goals, providing task-relevant feedback, and outlining the link between employee performance and rewards [29]. This form of leadership could provide a highly structured approach to help employees prepare for change by outlining the benefits of engaging in new practices to support the implementation of organizational change.

Nordin [28] conducted an empirical study of leadership and change readiness in the education sector and found that organizational commitment and transformational and transactional leadership behaviors explained 36.5% of the variance in readiness for change. These factors have also frequently been reported to support the implementation of health promotion initiatives in public health [30, 31] and hospital settings [32, 33]. Thus, it appears that organizational climate factors and the leadership behaviors of management staff affect organizational readiness for change.

While the factors described above have been found to be related to organizational readiness for change in the broader literature, predictors of organizational readiness for change in the aged care sector have not yet been examined. Given the potential investment in aged care facilities and impending changes within these facilities, this information is a vital key to assist change. The current study was conducted across a number of residential aged care facilities in Australia. It was designed to investigate how organizational culture and workplace practices are related to organizational readiness for change in the context of the introduction of training to ensure the provision of best practices in relation to the management of depression and Behavioral and Psychological Symptoms of Dementia (BPSD) [3]. First, it was hypothesized that organizational climate variables would be positively associated with organizational readiness for change. Second, it was hypothesized that, transformational and transactional leadership behaviors would be positively associated with organizational readiness for change above and beyond the contribution made by organizational climate.

## Methods

### Participants

The participants comprised 255 employees of 21 residential aged care facilities in Victoria, Australia. Of the participants, 222 (i.e., 87%) were female and 33 (i.e., 13%) were male. The age of participants ranged from 21 to 66 years (*M* = 42.86 years; *SD* = 12.13 years). Participants had worked in the aged care sector for a period of 6 months to 38 years (*M* = 10.86 years; *SD* = 8.70 years). The participants were grouped into two categories. The first group comprised employees in management roles (*n* = 131), including Registered Nurses (RNs), staff in managerial positions, physiotherapists, and staff who held multiple roles within their organization (e.g., one participant was a psychiatric nurse and an education manager). The second group comprised non-management staff, including Personal Care Assistants (PCAs) or Direct Carers (*n* = 124) who attend to the daily living activities of aged care residents (e.g., showering, dressing, and feeding) and other respondents such as leisure and lifestyle staff, an administration manager, and a cleaner.

### Materials

The study used several established instruments (described further below). For each of the established measures, the internal consistency (i.e., reliability) is reported through the calculation of Cronbach’s alpha (α). The data collected also included demographic measures such as gender, age, role(s) in the organization, and the number of years each participant had worked in the aged care sector.

Organizational climate was measured using the Organizational Climate Questionnaire (OCQ) [18]. The 40-item scale required participants to respond using a five-point Likert scale ranging from one (strongly disagree) to five (strongly agree) and included items such as “I make most of the decisions that affect the way my job is performed.” The scale comprised eight subscales (autonomy, cohesion, trust, work pressure, support, recognition, fairness, and innovation). The subscale reliabilities for the OCQ in the current study had good internal consistency (αs ≥ .80).

The Multifactor Leadership Questionnaire (MLQ) developed by Bass and Avolio [34] was adapted for this study and used to assess transformational and transactional leadership. Participants used a five-point Likert scale ranging from zero (not at all) to four (frequently, if not always) to indicate the frequency to which each item applied to them. Nine items (with an α = .91 in the current study) measured transformational leadership and included items such as “I enable others to look at problems in new ways” and nine items (with an α = .88 in the current study) measured transactional leadership and included items such as “I help keep others focused on the task at hand.”

The scale Readiness for Organizational Change was used to assess the readiness of individual employees for organizational change [15]. The four dimensions of readiness were: i) appropriateness (10 items) (i.e., whether staff members considered the change appropriate for the organization and were of the view that there was a valid need for a change; ii) change efficacy (six items) (i.e., whether staff members had confidence in their abilities to change); iii) management support (six items) (i.e., whether the leaders in the organization were actually committed to the change); and iv) personal valence (three items) (i.e., whether the change was believed to be personally beneficial). Participants recorded their level of agreement with each item using a five-point Likert scale ranging from one (strongly disagree) to five (strongly agree). These subscales had very good internal consistency (αs ≥ .89). See Additional file 1 to see the complete survey. 

### Procedure

Approval to conduct the study was granted by the University Human Research Ethics Committee. Either the research, training, or education managers of approximately 34 residential aged care providers were first contacted via email and then telephoned and asked if their staff could participate in the study. Only facilities with a minimum of sixty residents were approached. Eleven aged care providers, responsible for 21 facilities located in metropolitan Melbourne and rural Victoria, Australia agreed to allow their staff participate in the study. The recruitment of staff at each individual facility commenced upon organizational consent being obtained from the facility manager. Hard copy surveys were provided to staff at the facilities.

### Data analysis

Data analysis involved both preliminary and primary analysis. The preliminary analysis involved conducting a series of means difference tests between management and non-management staff on the variables included in the primary analysis – namely the subscales of the organizational climate and organizational readiness for change questionnaire, and both transformation and transactional leadership behaviors. The primary analysis consisted of conducting a hierarchical multiple regression to determine the contribution of the predictor variables of organizational climate and leadership upon the dependent variable organizational readiness for change. The hierarchical regression comprised two steps. All organizational climate variables (i.e., pressure, trust, innovation, recognition, cohesion, autonomy, fairness, and support) were entered at step one while the leadership variables (i.e., transformational and transactional leadership) were entered at step two.

## Results

The means and standard deviations for years working, eight organizational climate factors, two leadership behaviors, and organizational readiness for change are presented in Table 1. For organizational climate variables, staff reported moderate (and largely similar) levels of autonomy, cohesion, pressure, trust, support, recognition, fairness, and innovation within their organizations. Staff also reported moderate levels of transformational leadership and low levels of transactional leadership within their organizations, but relatively high levels of organizational readiness for change.

**Table 1:** Means and standard deviations of independent and dependent variables

	Managerial Staff (*n* = 131)		Non-Managerial Staff (*n* = 124)		Overall		
Variable	*M*	*SD*	*M*	*SD*	*M*	*SD*	Scale Range
Years Working					10.86	8.70	.5–38
Autonomy	3.84	.68	3.50	.75	3.66	.73	1–5
Cohesion	3.71	.72	3.56	.70	3.63	.71	1–5
Pressure	3.00	.78	2.81	.66	2.94	.74	1–5
Trust	3.50	.64	3.59	.77	3.54	.71	1–5
Support	3.48	.71	3.52	.72	3.50	.71	1–5
Recognition	3.41	.62	3.41	.71	3.41	.67	1–5
Fairness	3.59	.62	3.69	.67	3.64	.64	1–5
Innovation	3.69	.70	3.65	.80	3.67	.75	1–5
Transformational leadership	3.21	.64	2.97	.64	3.09	.58	1–5
Transactional leadership	2.92	.52	2.57	.72	2.76	.64	1–5
Organizational readiness	4.45	.44	4.28	.42	4.37	.43	1–5

There were few differences between management and non-management staff across organizational factors, leadership behavior, and organizational readiness for change. However, compared to senior staff, junior staff reported significantly higher autonomy (*M* = 3.50 versus 3.84, *t*[253] = − 3.67, *p* < .001) and transactional leadership behavior (*M* = 2.57 versus 2.92, *F*[253] = − 4.36, *p* < .001). Other differences were non-significant. As so few differences were found, it was deemed appropriate to collapse both staff groups within the one hierarchical regression analysis.

Step one of the hierarchical model was significant (*R* = .30. *R*^*2*^ = .15, *F*(8, 246) = 11.95, *p* < .001). The organizational climate variables of work pressure and innovation significantly contributed to the prediction of organizational readiness for change. At step two, an additional 8% of the variance in organizational readiness for change was accounted for (ΔR^2^ = .08, *R* = .48, *R*^*2*^ = .23, *F*(10, 244) = 7.36, *p* < .001). Transformational leadership (but not transactional leadership) significantly contributed to the model, and work pressure and innovation remained significant contributors. In total, 23% of the variance of readiness for change was accounted for by these variables (refer Table 2).

**Table 2:** Hierarchical multiple regression analyses of organizational readiness for change and leadership and organizational climate variables

Organizational Readiness for Change						
Step	Variables	B	S.E.	β	R^2^	ΔR^2^
1	Autonomy	.310	.192	.104	.15	
	Cohesion	−.117	.310	−.038		
	Trust	.005	.321	.002		
	Pressure	.667	.197	.225^**^		
	Support	.122	.316	.040		
	Recognition	.428	.284	.131		
	Fairness	.261	.322	.077		
	Innovation	.568	.244	.194^**^		
2	Autonomy	.172	.186	.058	.23	.08^*^
	Cohesion	.031	.297	.010		
	Trust	−.130	.299	−.042		
	Pressure	.522	.190	.176^**^		
	Support	.170	.302	.056		
	Recognition	.254	.273	.078		
	Fairness	.356	.311	.105		
	Innovation	.391	.240	.134^*^		
	Transformational Leadership	.741	.272	.272^**^		
	Transactional Leadership	.476	.476	.143		

^*^p < .05, ^**^p < .01

## Discussion

The present study sought to explore the role of organizational climate variables and leadership style in organizational readiness for change in aged care facilities. It was hypothesized that organizational climate variables (hypothesis 1) and leadership behaviors (hypothesis 2) would predict organizational readiness for change. In relation to hypothesis 1, two of the eight organizational climate factors (i.e., work pressure and innovation) were found to be significantly related to organizational readiness for change. Thus, our first hypothesis was partially supported. In relation to hypothesis 2, only transformational leadership behavior (and not transactional leadership behavior) was found to predict organizational readiness for change. Thus, our second hypothesis was also partially supported.

Given the lack of understanding of the contextual factors affecting the success of change in aged care environments [10] and the limited research that has been conducted in aged care facilities in relation to organizational readiness for change, a full suite of organizational climate factors was included in this study. The results of this study cannot be compared to other studies in aged care environments. However, it should be noted that innovation has previously been found to be a significant predictor of organizational readiness for change in other industry sectors [19]. Clearly, a more innovative work place is more likely to be ready for change. The findings of this study in relation to work pressure are novel, in that the relationship between work pressure and readiness for change was positive. One possibility is that workers perceive that any change will alleviate pressure, and consequently look forward or are ready for change to produce that positive outcome.

As stated above, the finding that transformational leadership predicted organizational readiness for change is consistent with previous research [25, 28]. This suggests that a transformational style of leadership is likely to engage and manage positive feelings and beliefs among employees in respect of organizational change. That transactional leadership style was not related to readiness for change suggests that adopting a task oriented and goal focused leadership approach is likely to be less effective in instilling a readiness for change among aged care staff than a transformational leadership approach. To ensure staff feel prepared for change, it is important that management staff instill a vision for the future and build the capacities of staff. Transactional leadership is largely devoid of these qualities; however, these qualities are inherent in transformational leadership. Transactional leadership may have a role in the implementation of change; however, it does not appear to have any role in preparing employees for change.

Collectively, these findings suggest that aged care facilities may have higher levels of success in implementing successful change initiatives when employees feel that their work environment is innovative and pressured, and managers adopt a transformational leadership style.

### Limitations and implications of findings

These findings might not be generalized to other aged care and health care settings. In the future, longitudinal research studies should be undertaken to validate the framework for organizational change. Further, it should be noted that only 23% of the variance in readiness for change was accounted for by the combined variables. Thus, various additional factors should be explored in future research.

The results of this study suggest that the success of change initiatives in residential aged care settings is enhanced by an organization’s capacity to create a work environment that promotes leadership practices aimed at increasing an organization’s readiness for change. Assessments of organizational readiness for change and the predictors identified in this study can be used to recognize organizational and workforce capacity and address any organizational limitations. Such knowledge could then be used to develop targeted interventions such as staff training aimed at changing practices in relation to the needs of residents. In addition to providing information about resident problems and related management strategies, any training should address work pressures, opportunities for innovation, and transformational leadership.

## Conclusion

This study examined the impact of organizational climate and leadership on organizational readiness for change in an aged care setting. Transformational leadership styles, an organizational climate of innovation, and work pressure were identified as being likely to assist organizations preparing for change. Any training program designed to change practices in aged care settings might incorporate these factors.

## Additional file

Additional file 1: Organisational Readiness for Changes in Aged Care Questionnaire (DOCX 102 kb)

## Abbreviations

MLQ: Multifactor Leadership Questionnaire
OCQ: Organizational Climate Questionnaire
PCA: Personal Care Assistants
RN: Registered Nurses
