# Supplementary material for: Organizational factors associated with readiness for change in residential aged care settings
Source: BMC Health Serv Res. 2018 Feb 1;18:77. doi: 10.1186/s12913-018-2832-4 (PMC5796299; doi:10.1186/s12913-018-2832-4)
Supplement: Additional file 1: — Organisational Readiness for Changes in Aged Care Questionnaire (DOCX 102 kb) [file 12913_2018_2832_MOESM1_ESM.docx]

SPSS TEMPLATE FOR PILOT MEASURES

**P1 = pre training (On our timeline chart this would be T1 = time one/ just before training)**

**P2 = post training (On our timeline chart this would be T2 = time two / just after training)**

**SCALE TITLES IN GREY BOXES**

**(Variable codes in red)**

**Numbers after variable codes relate to question numbers**

| 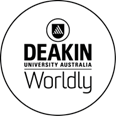  **RECORD SHEET OF AGED CARE STAFF**   1. **PARTICIPANT ID:_______________________________________**   **[Please use a personal code that is ONLY known to you – to ensure confidentiality e.g. mother’s maiden name + her date of birth e.g. skondras1938]**   1. **AGE (in years and months):________________________________** 2. **GENDER (please tick): □ male**   **□ female**   1. **ROLE DESCRIPTION (please tick):**   **□ Registered Nurse Div. 1**  **□ Registered Nurse Div. 2**  **□ Personal Care Assistant**  **□ Other – Please specify:______________**   1. **Management □**   **Team leader/Supervisor □**  **General employee □**   1. **NUMBER OF YEARS WORKING IN AGED CARE: _________**   **[Once you have completed this Pre/Post-training questionnaire, please place into the envelope provided, seal and hand to facilitator prior to Session 1 (or after completion of session 4) of the training program or forward to address provided on the replied paid envelope]** |
| --- |

**When you think about yourself and your job, how much of the time do you feel each of the following ways? Please circle the number that best described your feeling for the following statements**.

**WORK ENVIRONMENT
EMOTION (WEJobEmot)**

|  | Never | Once a month | A few times a month | Once a week | A few times a week | Almost every day |
| --- | --- | --- | --- | --- | --- | --- |
| 1. I feel emotionally drained by my job | 0 | 1 | 2 | 3 | 4 | 5 |
| 1. I feel burned-out by my job | 0 | 1 | 2 | 3 | 4 | 5 |
| 1. I feel frustrated at my job | 0 | 1 | 2 | 3 | 4 | 5 |
| 1. I feel tense at my job | 0 | 1 | 2 | 3 | 4 | 5 |
| 1. I lose my appetite because of my job-related problems | 0 | 1 | 2 | 3 | 4 | 5 |
| 1. Job-related problems keep me awake at night | 0 | 1 | 2 | 3 | 4 | 5 |
| 1. Job-related problems make my stomach upset | 0 | 1 | 2 | 3 | 4 | 5 |
| 1. Job-related problems make my heart beat faster than usual | 0 | 1 | 2 | 3 | 4 | 5 |

**In this section, please circle the appropriate number to indicate how satisfied or dissatisfied you are with various aspects of you job. Please answer each item.**

**WORK ENVIRONMENT – JOB SATISFACTION (WEJobSatisf)**

|  | Very Dissatisfied | Dissatisfied | Neither Satisfied nor Dissatisfied | Satisfied | Very Satisfied |
| --- | --- | --- | --- | --- | --- |
| 1. Job security (stable work) | 1 | 2 | 3 | 4 | 5 |
| 1. Physical conditions (lighting, ventilation, etc.) | 1 | 2 | 3 | 4 | 5 |
| 1. Fringe benefits (company discounts, superannuation, etc.) | 1 | 2 | 3 | 4 | 5 |
| 1. Pay you receive for your job | 1 | 2 | 3 | 4 | 5 |
| 1. The recognition you get when you do a good job | 1 | 2 | 3 | 4 | 5 |
| 1. The freedom you have to do the best you can at your job | 1 | 2 | 3 | 4 | 5 |
| 1. Your advancement to better positions since you started working for this organisation | 1 | 2 | 3 | 4 | 5 |
| 1. The work you do | 1 | 2 | 3 | 4 | 5 |

**Please circle the number to indicate whether** **you disagree or agree with each statement**:

**ORGANISATION CLIMATE QUESTIONNAIRE (OCQ)**

|  | Strongly Disagree | Disagree | Uncertain | Agree | Strongly Agree |
| --- | --- | --- | --- | --- | --- |
| 1. I make the most of the decisions that effect the way my job is performed | 1 | 2 | 3 | 4 | 5 |
| 1. I determine my own work procedures | 1 | 2 | 3 | 4 | 5 |
| 1. I schedule my own work activities | 1 | 2 | 3 | 4 | 5 |
| 1. I set the performance standards for my job | 1 | 2 | 3 | 4 | 5 |
| 1. I organise my work as I see best. | 1 | 2 | 3 | 4 | 5 |
| 1. People pitch in to help each other out. | 1 | 2 | 3 | 4 | 5 |
| 1. People tend to get along with each other | 1 | 2 | 3 | 4 | 5 |
| 1. People take a personal interest in one another | 1 | 2 | 3 | 4 | 5 |
| 1. There is a lot of “team spirit” among people at my work | 1 | 2 | 3 | 4 | 5 |
| 1. I feel like I have a lot in common with the people I know at my work | 1 | 2 | 3 | 4 | 5 |
| 1. I can count on my work colleagues to keep the things I tell them confidential | 1 | 2 | 3 | 4 | 5 |
| 1. My work colleagues have a lot of personal integrity | 1 | 2 | 3 | 4 | 5 |
| 1. My work colleagues are the kind of people I can level with | 1 | 2 | 3 | 4 | 5 |
| 1. My work colleagues are not likely to give me bad advice | 1 | 2 | 3 | 4 | 5 |
| 1. My work colleagues keep their commitments | 1 | 2 | 3 | 4 | 5 |
| 1. I have too much work and too little time to do it in | 1 | 2 | 3 | 4 | 5 |
| 1. My work is a relaxed place to work | 1 | 2 | 3 | 4 | 5 |
| 1. At home, I sometimes dread hearing the telephone ring because it might be someone calling about a job-related problem | 1 | 2 | 3 | 4 | 5 |
| 1. I feel like I never have a day off | 1 | 2 | 3 | 4 | 5 |
| 1. Too many employees at my level at work get “burned out” by the demands of their jobs | 1 | 2 | 3 | 4 | 5 |
| 1. I can count on my work colleagues to help me when I need it | 1 | 2 | 3 | 4 | 5 |
| 1. My work colleagues are interested in me getting ahead in the company | 1 | 2 | 3 | 4 | 5 |
| 1. My work colleagues are behind me 100%. | 1 | 2 | 3 | 4 | 5 |
| 1. My work colleagues are easy to talk to about job-related problems | 1 | 2 | 3 | 4 | 5 |
| 1. My work colleagues back me up and lets me learn from my mistakes | 1 | 2 | 3 | 4 | 5 |
| 1. I can count on a pat on the back when I perform well | 1 | 2 | 3 | 4 | 5 |
| 1. The only time I hear about my performance is when I screw up | 1 | 2 | 3 | 4 | 5 |
| 1. My work colleagues know what my strengths are and let me know it | 1 | 2 | 3 | 4 | 5 |
| 1. My work colleagues are quick to recognise good performance | 1 | 2 | 3 | 4 | 5 |
| 1. My work colleagues use me as an example of what to do | 1 | 2 | 3 | 4 | 5 |
| 1. I can count on a fair go from my work colleagues | 1 | 2 | 3 | 4 | 5 |
| 1. The objectives my boss sets for my job are reasonable | 1 | 2 | 3 | 4 | 5 |
| 1. My boss is not likely to give me a bad deal | 1 | 2 | 3 | 4 | 5 |
| 1. My boss does not play favourites | 1 | 2 | 3 | 4 | 5 |
| 1. If my boss terminates someone, the person probably deserved it | 1 | 2 | 3 | 4 | 5 |
| 1. My boss encourages me to develop my ideas | 1 | 2 | 3 | 4 | 5 |
| 1. My boss likes me to try new ways of doing my job | 1 | 2 | 3 | 4 | 5 |
| 1. My boss encourages me to improve on his/her methods | 1 | 2 | 3 | 4 | 5 |
| 1. My boss encourages me to find new ways around old problems | 1 | 2 | 3 | 4 | 5 |
| 1. My boss “talks up” new ways of doing things | 1 | 2 | 3 | 4 | 5 |

**We would like to know how your immediate supervisor helps you when you face job-related problems. Please answer the following questions by circling the most appropriate number.**

**WORK ENVIRONMENT**

**(WEProb)**

|  | Not at all | A little | Somewhat | Very much |
| --- | --- | --- | --- | --- |
| 1. How much does your immediate supervisor go out of his/her way to make your work-life easier for you? | 1 | 2 | 3 | 4 |
| 1. How easy is it for you to talk with your immediate supervisor about your job-related problems? | 1 | 2 | 3 | 4 |
| 1. How much can your immediate supervisor be relied on when things get tough at your job? | 1 | 2 | 3 | 4 |

**The items below relate to how you currently communicate with work colleagues in your organisation. Rate to what extent you think the item best reflects your CURRENT communication with others using the following scale:**

**COMMUNICATION SCALE**

**(Comm)**

| **At this point in time in my organisation…** | Almost never behave this way | Seldom behave this way | Sometimes behave this way | Often behave this way | Almost always behave this way |
| --- | --- | --- | --- | --- | --- |
| 1. I allow work colleagues to see who I really am | 1 | 2 | 3 | 4 | 5 |
| 1. Work colleagues know what I’m thinking | 1 | 2 | 3 | 4 | 5 |
| 1. I reveal how I feel to work colleagues | 1 | 2 | 3 | 4 | 5 |
| 1. When I’ve been wronged, I confront the work colleague who wronged me | 1 | 2 | 3 | 4 | 5 |
| 1. I have trouble standing up for myself in the workplace | 1 | 2 | 3 | 4 | 5 |
| 1. I stand up for my rights in the workplace | 1 | 2 | 3 | 4 | 5 |
| 1. I let work colleagues know that I understand what they say | 1 | 2 | 3 | 4 | 5 |
| 1. In the workplace, I take charge of conversations I’m in by negotiating what topics we talk about | 1 | 2 | 3 | 4 | 5 |
| 1. It’s difficult to find the right words to express myself in the workplace | 1 | 2 | 3 | 4 | 5 |
| 1. I express myself well verbally in the workplace | 1 | 2 | 3 | 4 | 5 |
| 1. My communication is usually descriptive not evaluative in the workplace | 1 | 2 | 3 | 4 | 5 |
| 1. I communicate with work colleagues as though they’re my equals | 1 | 2 | 3 | 4 | 5 |
| 1. Other work colleagues would describe me as warm | 1 | 2 | 3 | 4 | 5 |
| 1. My work colleagues truly believe that I care about them | 1 | 2 | 3 | 4 | 5 |
| 1. I have trouble convincing work colleagues to do what I want them to do | 1 | 2 | 3 | 4 | 5 |

**This questionnaire relates to how often you CURRENTLY perform the following workplace behaviours when working with colleagues**

**TRANSFORMATIONAL / TRANSACTIONAL ITEMS (Trans)**

| **At this point in time…….** | Not at all | Once in a while | Sometimes | Fairly often | Frequently if not always |
| --- | --- | --- | --- | --- | --- |
| 1. I make others feel enthusiastic about learning tasks and assignments | 0 | 1 | 2 | 3 | 4 |
| 1. I express appreciation if someone does a good job | 0 | 1 | 2 | 3 | 4 |
| 1. I give personal attention to others who seem neglected | 0 | 1 | 2 | 3 | 4 |
| 1. I enable others to look at problems in new ways | 0 | 1 | 2 | 3 | 4 |
| 1. I help keep others working to capacity | 0 | 1 | 2 | 3 | 4 |
| 1. I help keep people working in terms co-ordinated | 0 | 1 | 2 | 3 | 4 |
| 1. I communicate to others what is expected of them | 0 | 1 | 2 | 3 | 4 |
| 1. I decide for others what should be done and how it will be done | 0 | 1 | 2 | 3 | 4 |
| 1. I help keep the others focused on the task at hand | 0 | 1 | 2 | 3 | 4 |

**The following items relate to your CURRENT perceptions of your work colleagues. That is, the extent that you perceive your work colleagues at this point in time.**

**TRUST SCALE (Trust)**

| **At this point in time….** | Not at all | Once in a while | Sometimes | Fairly often | Frequently if not always |
| --- | --- | --- | --- | --- | --- |
| 1. I can count on my work colleagues to be concerned about my welfare. | 0 | 1 | 2 | 3 | 4 |
| 1. I am familiar with work colleagues’ patterns of behaviour and can rely on them to behave in certain ways. | 0 | 1 | 2 | 3 | 4 |
| 1. Even when I don't know how my work colleagues will react, I feel comfortable telling them anything about workplace behaviour; even those things of which I am ashamed. | 0 | 1 | 2 | 3 | 4 |
| 1. Though times may change and the future is uncertain; I know my work colleagues will always be ready and willing to offer me strength and support. | 0 | 1 | 2 | 3 | 4 |
| 1. 1 am never certain that my work colleagues won’t do something that I dislike or will embarrass me. | 0 | 1 | 2 | 3 | 4 |
| 1. My work colleagues are very unpredictable. I never know how they are going to act from one day to the next. | 0 | 1 | 2 | 3 | 4 |
| 1. I feel very uncomfortable when my work colleagues have to make decisions which will affect me personally. | 0 | 1 | 2 | 3 | 4 |
| 1. My work colleagues behave in a very consistent manner. | 0 | 1 | 2 | 3 | 4 |
| 1. I can rely on my work colleagues to react in a positive way when I expose my weaknesses to them. | 0 | 1 | 2 | 3 | 4 |
| 1. When I share my problems with my work colleagues, I know they will respond in a supportive way even before I say anything. | 0 | 1 | 2 | 3 | 4 |
| 1. As part of our working relationship I have to keep alert or my work colleagues might take advantage of me. | 0 | 1 | 2 | 3 | 4 |
| 1. I sometimes avoid my work colleagues because they are unpredictable and I fear saying or doing something which might create conflict. | 0 | 1 | 2 | 3 | 4 |
| 1. I can rely on my work colleagues to keep the promises they make to me. | 0 | 1 | 2 | 3 | 4 |
| 1. When I am with my work colleagues I feel secure in facing unknown new workplace situations. | 0 | 1 | 2 | 3 | 4 |
| 1. I am willing to let work colleagues make decisions for me. | 0 | 1 | 2 | 3 | 4 |

**The following items relate to your CURRENT perceptions of your organisation. That is, the extent that you perceive your organisation at this point in time.**

**Please circle the number to indicate whether you disagree or agree with each statement:**

**ORGANISATIONAL READINESS – FACTOR 1 APPROPRIATENESS (ORapp)**

| **At this point in time……** | Strongly Disagree | Disagree | Neither Agree nor Disagree | Agree | Strongly Agree |
| --- | --- | --- | --- | --- | --- |
| 1. I think that the organisation will benefit from this change | 1 | 2 | 3 | 4 | 5 |
| 1. It doesn’t make much sense for us to initiate this change | 1 | 2 | 3 | 4 | 5 |
| 1. There are legitimate reasons for us to make this change | 1 | 2 | 3 | 4 | 5 |
| 1. This change will improve our organisation’s overall efficiency | 1 | 2 | 3 | 4 | 5 |
| 1. There are a number of rational reasons for this change to be made | 1 | 2 | 3 | 4 | 5 |
| 1. In the long run, I feel it will be worthwhile for me if the organisation adopts this change | 1 | 2 | 3 | 4 | 5 |
| 1. This change makes my job easier | 1 | 2 | 3 | 4 | 5 |
| 1. When this change is implemented, I don’t believe there is anything for me to gain | 1 | 2 | 3 | 4 | 5 |
| 1. The time we are spending on this change should be spent on something else | 1 | 2 | 3 | 4 | 5 |
| 1. This change matches the priorities of our organisation | 1 | 2 | 3 | 4 | 5 |

**ORGANISATIONAL READINESS – FACTOR 4 – PERSONALLY BENEFICIAL (ORbenef)**

| **At this point in time….** | Strongly Disagree | Disagree | Neither Agree nor Disagree | Agree | Strongly Agree |
| --- | --- | --- | --- | --- | --- |
| 1. I am worried I will lose some of my status in the organisation when this change is implemented | 1 | 2 | 3 | 4 | 5 |
| 1. This change will disrupt many of the personal relationships I have developed | 1 | 2 | 3 | 4 | 5 |
| 1. My future in this job will be limited because of this change | 1 | 2 | 3 | 4 | 5 |

**ORGANISATIONAL READINESS – FACTOR 2 – MANAGEMENT SUPPORT**

**(ORsupp)**

| **At this point in time….** | Strongly Disagree | Disagree | Neither Agree nor Disagree | Agree | Strongly Agree |
| --- | --- | --- | --- | --- | --- |
| 1. Our senior leaders have encouraged all of us to embrace this change | 1 | 2 | 3 | 4 | 5 |
| 1. Our organisation’s top decision makers have put all their support behind this change effort | 1 | 2 | 3 | 4 | 5 |
| 1. Every senior manager has stressed the importance of this change | 1 | 2 | 3 | 4 | 5 |
| 1. This organisation’s most senior leader is committed to this change | 1 | 2 | 3 | 4 | 5 |
| 1. I think we are spending a lot of time on this change when the senior managers don’t even want it implemented | 1 | 2 | 3 | 4 | 5 |
| 1. Management has sent a clear signal this organisation is going to change | 1 | 2 | 3 | 4 | 5 |

**ORGANISATIONAL READINESS – FACTOR 3 – CHANGE EFFICACY**

**(ORefficacy)**

| **At this point in time….** | Strongly Disagree | Disagree | Neither Agree nor Disagree | Agree | Strongly Agree |
| --- | --- | --- | --- | --- | --- |
| 1. I do not anticipate any problems adjusting to the work I will have when this change is adopted | 1 | 2 | 3 | 4 | 5 |
| 1. There are some tasks that will be required when we change that I don’t think I can do well | 1 | 2 | 3 | 4 | 5 |
| 1. When we implement this change, I feel I can handle it with ease | 1 | 2 | 3 | 4 | 5 |
| 1. I have the skills that are needed to make this change work | 1 | 2 | 3 | 4 | 5 |
| 1. When I set my mind to it, I can learn everything that will be required when this change is adopted | 1 | 2 | 3 | 4 | 5 |
| 1. My past experiences make me confident that I will be able to perform successfully after this change is made | 1 | 2 | 3 | 4 | 5 |

**Now, we would like to know how you feel about working for this organization. Please circle the number that best describes your feeling for each of the following statements.**

**WORK ENVIRONMENT – VIEW**

**(WEviewsorg)**

|  | Strongly Disagree | Disagree | Neither Agree nor Disagree | Agree | Strongly Agree |
| --- | --- | --- | --- | --- | --- |
| 1. I will work harder than I have to in order to help this organisation to be successful | 1 | 2 | 3 | 4 | 5 |
| 1. I am proud to work for this organisation | 1 | 2 | 3 | 4 | 5 |
| 1. I feel very little loyalty to this organisation | 1 | 2 | 3 | 4 | 5 |
| 1. I talk about this organisation to my friends as a great organisation to work for | 1 | 2 | 3 | 4 | 5 |
| 1. I really care about the fate of this organisation | 1 | 2 | 3 | 4 | 5 |

**WORK ENVIRONMENT – INTENTION (WEIntent)**

**The following statements are related to your job performance and intention to stop working for this organization. Please answer each of the following items.**

| 1. How often do you think of leaving your present job? | Rarely or Never | Occasionally | Sometimes | Fairly Often | Very Often |
| --- | --- | --- | --- | --- | --- |
| 1. How likely are you to look for a new job within the next year? | Very Unlikely | Unlikely | Not Sure | Likely | Very Likely |
| 1. How often in the last year did you seek or apply for another job? | Rarely or Never | Occasionally | Sometimes | Fairly Often | Very Often |

***Thank you for taking the time to answer the questions?***
